# Rare functional genetic variants in *COL7A1*, *COL6A5*, *COL1A2* and *COL5A2* frequently occur in Chiari Malformation Type 1

**DOI:** 10.1371/journal.pone.0251289

**Published:** 2021-05-11

**Authors:** Aintzane Urbizu, Melanie E. Garrett, Karen Soldano, Oliver Drechsel, Dorothy Loth, Anna Marcé-Grau, Olga Mestres i Soler, Maria A. Poca, Stephan Ossowski, Alfons Macaya, Francis Loth, Rick Labuda, Allison Ashley-Koch

**Affiliations:** 1 Duke Molecular Physiology Institute, Duke University Medical Center, Durham, NC, United States of America; 2 Pediatric Neurology Research Group, Vall d’Hebron Research Institute, Barcelona, Spain; 3 Genomic and Epigenomic Variation in Disease Group, Centre for Genomic Regulation (CRG), The Barcelona Institute of Science and Technology, Barcelona, Spain; 4 Universitat Pompeu Fabra, Barcelona, Spain; 5 Department of Psychology, Conquer Chiari Research Center, University of Akron, Akron, OH, United States of America; 6 Neurotraumatology and Neurosurgery Research Unit, Vall d’Hebron Research Institute, Universitat Autònoma de Barcelona, Barcelona, Spain; 7 Department of Neurosurgery, Vall d’Hebron University Hospital, Universitat Autònoma de Barcelona, Barcelona, Spain; 8 Department of Biomedical Engineering, Conquer Chiari Research Center, University of Akron, Akron, OH, United States of America; 9 Department of Mechanical Engineering, Conquer Chiari Research Center, University of Akron, Akron, OH, United States of America; 10 Conquer Chiari, Wexford, PA, United States of America; German Cancer Research Center (DKFZ), GERMANY

## Abstract

Chiari Malformation Type 1 (CM-1) is characterized by herniation of the cerebellar tonsils below the foramen magnum and the presence of headaches and other neurologic symptoms. Cranial bone constriction is suspected to be the most common biologic mechanism leading to CM-1. However, other mechanisms may also contribute, particularly in the presence of connective tissue disorders (CTDs), such as Ehlers Danlos Syndrome (EDS). Accumulating data suggest CM-1 with connective tissue disorders (CTD+) may have a different patho-mechanism and different genetic risk factors than CM-1 without CTDs (CTD-). To identify CM-1 genetic risk variants, we performed whole exome sequencing on a single large, multiplex family from Spain and targeted sequencing on a cohort of 186 unrelated adult, Caucasian females with CM-1. Targeted sequencing captured the coding regions of 21 CM-1 and EDS candidate genes, including two genes identified in the Spanish family. Using gene burden analysis, we compared the frequency of rare, functional variants detected in CM-1 cases versus publically available ethnically-matched controls from gnomAD. A secondary analysis compared the presence of rare variants in these genes between CTD+ and CTD- CM-1 cases. In the Spanish family, rare variants co-segregated with CM-1 in *COL6A5*, *ADGRB3* and *DST*. A variant in *COL7A1* was present in affected and unaffected family members. In the targeted sequencing analysis, rare variants in six genes (*COL7A1*, *COL5A2*, *COL6A5*, *COL1A2*, *VEGFB*, *FLT1*) were significantly more frequent in CM-1 cases compared to public controls. In total, 47% of CM-1 cases presented with rare variants in at least one of the four significant collagen genes and 10% of cases harbored variants in multiple significant collagen genes. Moreover, 26% of CM-1 cases presented with rare variants in the *COL6A5* gene. We also identified two genes (*COL7A1*, *COL3A1*) for which the burden of rare variants differed significantly between CTD+ and CTD- CM-1 cases. A higher percentage of CTD+ patients had variants in *COL7A1* compared to CTD+ patients, while CTD+ patients had fewer rare variants in *COL3A1* than did CTD- patients. In summary, rare variants in several collagen genes are particularly frequent in CM-1 cases and those in *COL6A5* co-segregated with CM-1 in a Spanish multiplex family. *COL6A5* has been previously associated with musculoskeletal phenotypes, but this is the first association with CM-1. Our findings underscore the contribution of rare genetic variants in collagen genes to CM-1, and suggest that CM-1 in the presence and absence of CTD symptoms is driven by different genes.

## Introduction

Chiari Malformations (CMs) are a group of malformations characterized by a downward herniation of the caudal part of the cerebellum and/or medulla oblongata into the spinal canal. Chiari Malformation Type 1 (CM-1) is the most common form of CMs, and is typically diagnosed as cerebellum tonsillar herniation (TH) of 3mm or more below the foramen magnum using magnetic resonance imaging (MRI) [1]. Clinically, CM-1 frequently presents with headache, ocular disturbances, vertigo, sleep apnea, lower cranial nerve signs, and motor and sensory symptoms. The symptoms are thought to result from a direct compression of neural tissue at the craniocervical junction and can also include cerebrospinal fluid disturbances, such as syringomyelia. Importantly, CM-1 individuals meeting diagnostic criteria on imaging can also be asymptomatic. TH in CM-1 is typically attributed to a cranial constriction mechanism caused by a small posterior cranial fossa (PCF) and concomitant shorter occipital bone. This mechanism may be caused by an insufficient paraxial mesoderm (classical CM-1) [2], premature synchondrosis closure, premature fusion of the skull bones (craniosynostosis) or a consequence of other conditions such as acromegaly or Paget’s disease [3]. However, additional mechanisms can also lead to TH, such as space-occupying intracranial lesions, cranial settling or tethered cord syndrome [3]. The other forms of CMs are generally less common and it remains unclear whether the biologic mechanisms leading to other CMs are related to those underlying CM-1.

Even within CM-1, there is clinical complexity. CM-1 is often comorbid with other conditions, especially connective tissue disorders (CTDs). In particular, CM-1 frequently co-occurs with Ehlers-Danlos Syndrome (EDS), a heterogeneous group of heritable CTDs characterized mainly by skin hyperextensibility, abnormal wound healing, and joint hypermobility [4, 5]. There are thirteen EDS subtypes caused by mutations in collagen-encoding genes or in genes encoding collagen-modifying enzymes [6]. CM-1 can also be comorbid with other CTD genetic syndromes, such as Marfan syndrome and Klippel Feil syndrome [7, 8]. Importantly, comorbidity with CTDs is also associated with differences in cranial morphology [5], suggesting that the comorbidity represents distinct clinical subtypes of CM-1.

The co-segregation of CM-1 with other known genetic conditions also suggests that genetic factors contribute to risk for CM-1. However, even in the absence of other comorbid conditions, several lines of evidence support a genetic basis for CM-1 risk. Between 12–20% of individuals with CM-1 report having a relative who has also been diagnosed with CM-1 or has similar clinical symptoms [9]. Several families comprised of multiple individuals with CM-1 (multiplex families) have been described, some presenting with complete or incompletely penetrant autosomal dominant inheritance and others with autosomal recessive inheritance [9–14]. Monozygotic twins who are concordant for CM-1 have also been observed [7]. Together, these findings strongly argue for a genetic contribution to CM-1 pathogenicity in at least a subset of cases. Despite this, the precise genetic variants contributing to CM-1 remain elusive in most cases.

Identifying the genetic risk factors for CM-1 can improve the understanding of the mechanistic processes that lead to the condition, but also provide the opportunity for genetic testing to families and individuals and ultimately, the possibility of therapeutic intervention. Several genetic approaches (linkage analysis, genetic association, expression analysis, sequencing studies) have been applied to CM-1. These studies have implicated genes involved in a multitude of biologic processes, including bone development and differentiation, angiogenesis and vasculogenesis, components of the extracellular matrix (ECM) and connective tissue, cell proliferation and differentiation, and ribosomal proteins [8, 11, 12, 15–18]. Most of the findings from these studies have been limited to single cohorts, with little or no evidence for replication. In short, a wide array of cellular and developmental processes may play a role in risk for CM-1, making the task of finding the relevant genes all the more challenging. Reducing phenotypic heterogeneity, and purportedly, the underlying genetic heterogeneity, can improve the ability to detect associated genetic factors. For example, stratifying families and patient cohorts on the presence of CTD-related symptoms or MRI-morphometric traits has successfully uncovered genetic variants that were not identified when analyzing all families or patients together [12, 15–17].

In recent years, genetic and genomic technologies have shifted towards next generation sequencing (NGS) approaches. Application of these approaches to CM-1 could improve the identification of causal variants in family and cohort studies. Exome sequencing has previously been applied to two Italian families with CM-1 resulting in three potential candidates, including *DKK1*, *LRP4* and *BMP1* [19]. The mutations in those genes co-segregated in the two families with CM-1, but screening 65 unrelated additional CM-1 probands only identified one additional missense mutation in *DKK1*. Another study performed exome sequencing on seven large multiplex Russian CM-1 families and linkage analysis suggested two chromosomal regions on 1q43-44 and 12q23-24.11, but the actual gene(s) and variants responsible were not definitively identified [14]. These findings highlight the potential power of NGS in identifying CM-1 genes, but also underscore the extensive genetic heterogeneity and the challenges for replicating genetic findings for this condition.

Here, we have utilized NGS technology to identify rare, putative causal genetic variants, and explored genetic heterogeneity as a function of comorbid symptoms consistent with CTD. We first performed whole exome sequencing in a Spanish family with multiple CM-1 affected individuals in an attempt to increase the likelihood of identifying at least one CM-1 gene. Next, we performed targeted sequencing in a US Caucasian cohort of the most promising candidate genes identified in the Spanish family, as well as several other genes previously associated with CM-1 and EDS. Our findings implicate several extracellular matrix and bone development genes in risk for CM-1, and suggest that rare variants in collagen genes are commonly present in CM-1.

## Results

### WES analysis of Spanish multiplex family

**Fig 1:**
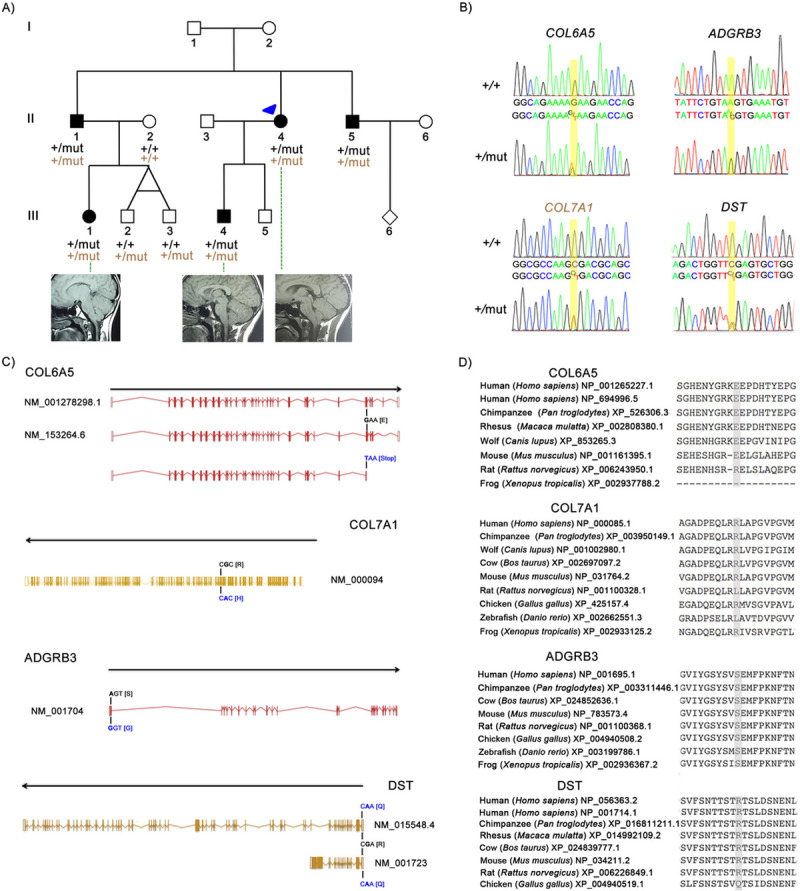
Mutations identified in the multiplex family with CM-1. (A) Pedigree of the Spanish family including five individuals with CM-1 carrying the heterozygous mutations E2272* in *COL6A5*, S45G in *ADGRB3*, R22Q in *DST*, in the top row; and R1202H in *COL7A1* in the bottom row. The arrow head indicates the index case (individual II.4). Healthy individuals are denoted in white and CM-1 affected in black. (B) Electropherograms showing the altered nucleotides (+/mut) and the respective wild type (+/+) sequences. (C) Location and consequence of the alteration in the human transcripts. (D) Evolutionary conservation of the E2272*, R1202H, S45G and R22Q residues in different species demonstrated by protein sequence alignment generated by MUSCLE version 3.6 (using option:-maxiters 2).

Variant detection from the whole exome sequencing data performed in four CM-1 affected members (see Fig 1, individuals II.1, II.4, III.1 and III.4) of a Spanish multiplex family yielded 974,936 total variants. After filtering as described in Methods, 2516 candidate variants remained (exonic and UTR). Among those, 1580 variants were annotated as affecting the protein sequence (nonsynonymous or splicing). We excluded variants with a MAF>0.01 (n = 1495), those where the functional prediction was neutral, tolerant or benign in five or more of the six prediction programs considered (n = 43), or those not exhibiting conservation across 20 different mammalian species (n = 18). Among the remaining 24 variants, seven were in pathways involved in angiogenesis (*ADGRB3*, *ADGRA2*) or the extracellular matrix (*COL6A5*, *COL7A1*, *DST*, *COL15A1*, *ITIH5*) (Table 1) and were confirmed by Sanger sequencing. Variants located in *COL6A5*, *ADGRB3* and *DST* were also present in the remaining affected sibling (individual II.5), but not in other family members who were not diagnosed with CM-1 (II.2, III.2 and III.3), suggesting cosegregation with the disease in the family (Fig 1A and 1B). The variant located in *COL7A1*, however, was present not only in the affected members but also in the twins who were not diagnosed with CM-1 (II.5, III.2, and III.3; Fig 1A and 1B), suggesting this variant could be incompletely penetrant. Variants located in *ADGRA2*, *COL15A1*, *ITIH5* were not present in the other affected sibling (individual II.5; data not shown), and thus were removed from consideration.

**Table 1:** Single nucleotide variations identified in the family with five CM-1 affected members when considering a dominant inheritance model.

Position (GRCh37/hg19)	Gene	Ref	Alt	dbsnp.b141	Impact variant	PP2	Shift	Mutation Taster	ExAC ENF	1000G EUR	GoESP6500SI
chr3:48623625	*COL7A1*	C	T	rs149011081	NS	D	D	D	0.0029	0.0030	0.0030
chr3:130187662	*COL6A5*	G	T	rs115375867	Stop gained	-	-	-	0.0112	0.0010	0.0070
chr6:69348700	*ADGRB3*	A	G	rs138295002	NS	B	T	D	0.0005	0.0010	0.0010
chr6:56507522	*DST*	C	T	rs749722200	NS	D	D	-	0.0001	-	-
chr8:37693279	*ADGRA2*	G	A	rs77369926	NS	D	T	D	0.0058	0.0050	0.0030
chr9:101816909	*COL15A1*	A	G	rs35544077	NS	T	D	D	0.0088	0.0050	0.0080
chr10:7628008	*ITIH5*	G	A	-	NS	D	D	D	-	-	-

NS (nonsynonymous), D (deleterious), B (benign), T (tolerant)

The variant located in *COL6A5* (chr3:130468818) is present in two isoforms (NM_001278298.1: c.6814G>T for transcript 1 and NM_153264.6: c.6814G>T for transcript 2) and leads to a stopgain: p.Glu2272* in both precursors (NP_001265227.1 and NP_694996.5) (Fig 1C). The affected amino acid position 2272 in the COL6A5 protein is conserved across different mammalian species, but not in amphibians (frog) where the protein is shorter (2296 amino acids) (Fig 1D). The variant located in *COL7A1* (chr3:48586192): NM_000094:c.G3605A leads to an amino acid change: p.Arg1202His (NP_000085.1) (Fig 1C), producing a hydrophilicity change from hydrophilic to moderate, but not a change in charge. The affected amino acid position 1202 in the COL7A1 protein is conserved in most mammals (except in rat), birds (such as chicken) and amphibians (frog) (Fig 1D). The missense variant in *ADGRB3* (chr6: 68638808): NM_001704:c.A133G leads to an amino acid change, p.Ser45Gly (NP_001695.1) (Fig 1C), and the hydrophilicity changes from polar to hydrophobic. This amino acid is conserved in mammals, birds (such as chicken), fish (zebrafish) and amphibians (frog) (Fig 1D). However, PolyPhen2 and Shift predicted the variant to be benign and tolerant, respectively (Table 1). The variant located in *DST* (chr 6: 56642724) impacts multiple transcripts of the gene: NM_001723:c.65G>A for transcript 1e and NM_015548.4:c.65G>A for transcript variant 1eA (Fig 1C). The variant leads to a missense mutation p.Arg22Gln in both isoforms of the protein (NP_001714.1 and NP_056363.2, respectively) and produces a loss of charge. This amino acid is conserved in some isoforms of human, chimpanzee and mouse, but not in birds (such as chicken), fish (zebrafish) or amphibians (frog) (Fig 1D). Based on these data, the functional consequence of the *COL6A5* variant (stopgain) appeared to be the most significant, but we were unable to conclusively rule out any of the other variants. Thus, we sought to evaluate these and other, related genes in an independent cohort.

### Network protein-protein interaction

**Fig 2:**
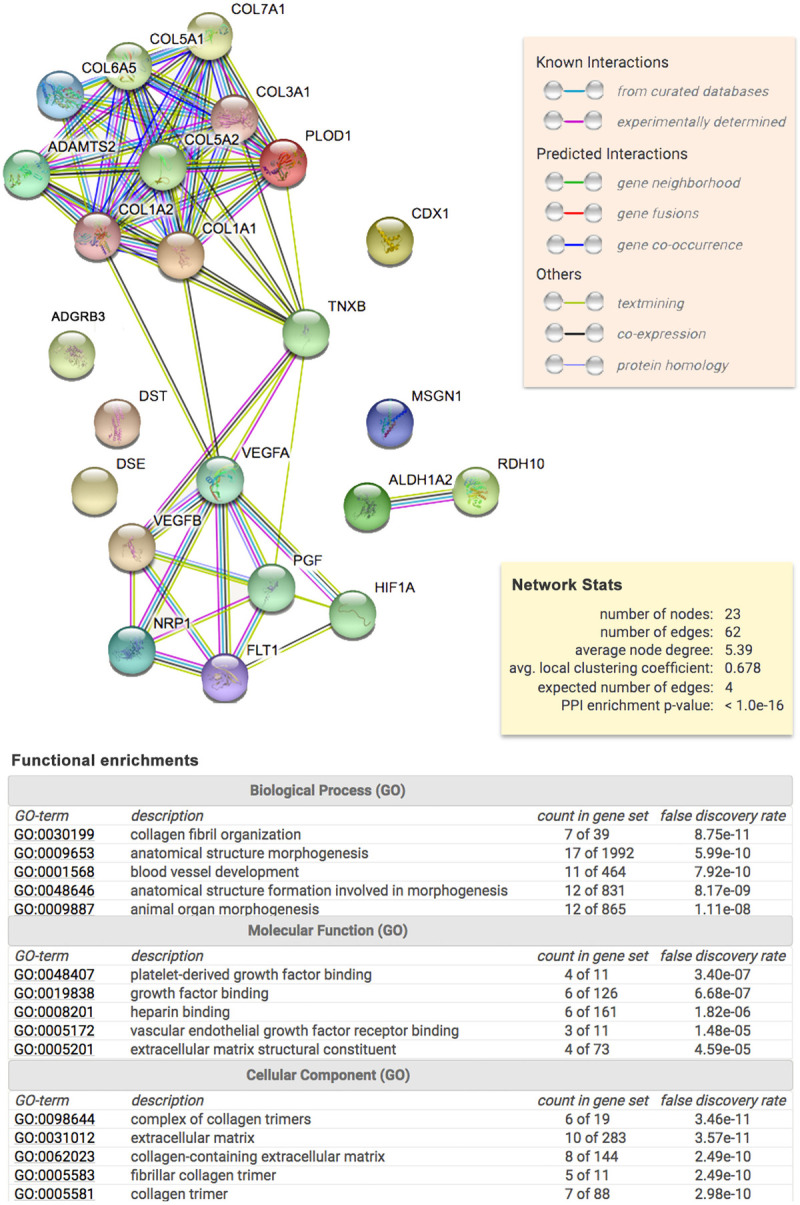
Protein network and enrichment analysis obtained from STRING. In the upper part, the diagram shows the result obtained upon entering a set of 23 proteins suspected to be involved in CM-1. Each node represents all the proteins produced by a single, protein-coding gene locus. Colored nodes indicate query proteins and first set of interactions. Filled node content indicates that protein 3D structure is known/predicted. Edges represent specific and meaningful protein-protein associations. In the middle, the yellow rectangular shows the reported enrichment of functional connections among the set of proteins. At the bottom, the table shows the statistical enrichment detected in functional subsystems.

Since variants in two collagens (*COL6A5* and *COL7A1*) were detected in the Spanish family, we hypothesized that a common pathway of collagens (including EDS genes) could be involved in CM-1 pathogenesis. Thus, in the targeted sequencing analysis, we considered *COL6A5* and *COL7A1*, as well as *COL1A2*, *COL5A1*, *COL5A2*, *COL1A1*, *ADAMTS2*, *TNXB* and *PLOD1*. We also considered five genes that were previously described to be associated with CM-1: *ALDH1A2*, *CDX1*, *FLT1*, *MSGN1*, and *RDH10*. Those genes were input into the STRING protein network database to identify a potential protein-protein interaction (PPI) network for CM-1. Seven other genes were ultimately included (*COL3A1*, *DSE*, *HIF1A*, *NRP1*, *PGF*, *VEGFA* and *VEGFB)* to create a fully connected network. The PPI enrichment was statistically significant (p<1.0e-16), meaning that these proteins have more interactions among themselves than what would be expected for a random set of proteins of similar size. Such enrichment suggests that the proteins are biologically connected. Through this STRING analysis, we determined that *ADGRB3* and *DST* were not connected to this network, even after extending the interaction connections five times. Thus, we omitted those two genes from the targeted sequencing analysis and only examined the remaining 21 ECM genes belonging to the collagen/EDS network (Fig 2).

### Targeted sequencing

A total of 1833 variants were observed across the samples subjected to targeted sequencing. Two samples had low coverage (<10X) and were removed. After zeroing out variants called from fewer than 3 reads, three additional samples had missing rates > 50%, and therefore were removed from the analysis. Two samples were removed because the average number of variants detected was > 3 standard deviations from the mean, likely indicating a sample quality issue. Finally, one sample had over half of the total variants called as homozygous alternate, again, indicating a sample quality issue and thus, it was also removed. After all quality control measures, 178 CM-1 cases (average coverage of 143X) and 1345 variants (777 of which were exonic and 489 of which were functional) were included in the subsequent analyses. Of note, three of the candidate genes (*ALDH1A2*, *CDX1* and *RDH10*) were also excluded from analysis at this point because these genes did not contain variants that passed the quality control filters.

**Fig 3:**
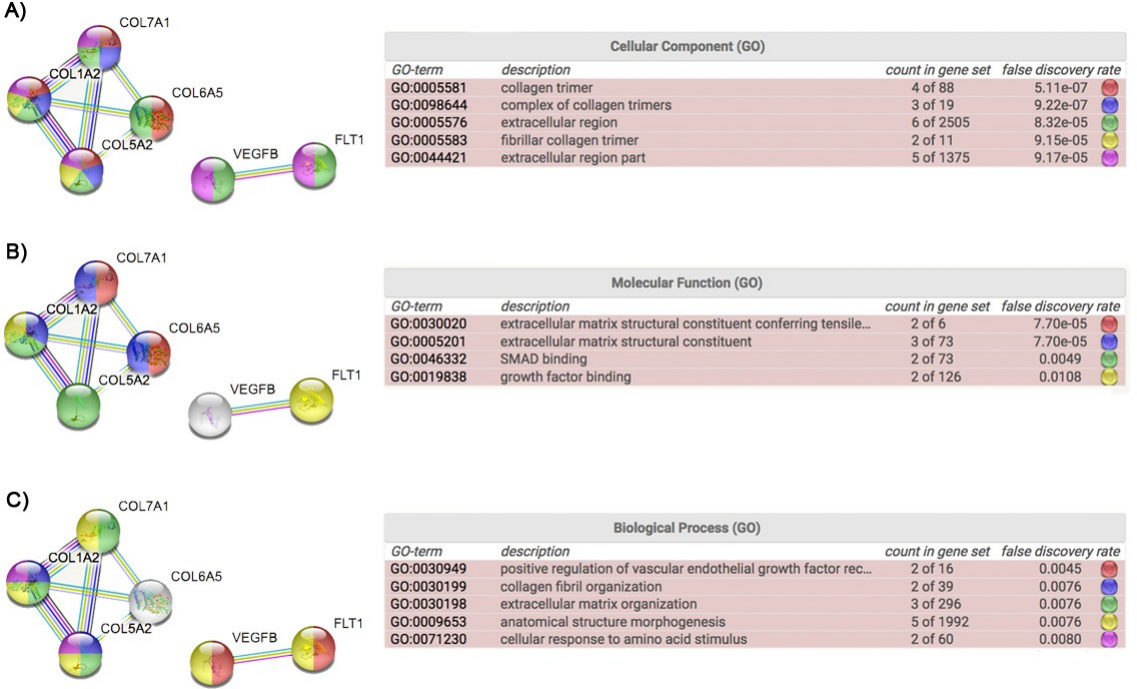
Protein enrichment analysis obtained from STRING for the six genes with significantly more rare variants in CM-1 compared with controls: *COL5A2*, *COL6A5*, *COL1A2*, *COL7A1*, *VEGFB* and *FLT1*. (A), (B) and (C) show the results obtained from the cellular component, molecular function and biological process enrichment, respectively.

To compare rare, functional variant allele frequencies in CM-1 cases and controls (publically available from the gnomAD database), we restricted the analysis to only missense variants with MAF<0.05. We identified six genes (*COL7A1*, *COL5A2*, *COL6A5*, *COL1A2*, *VEGFB* and *FLT1*) with a higher burden of rare, functional variants in CM-1 cases compared to controls (p’s < 0.01; Tables 2 and S3) with odds ratios ranging from 1.8 (*COL5A2*) to 8.3 (*COL1A2*) (Table 2). STRING (PPI) enrichment showed that these proteins are at least partially biologically connected (p = 3.49e-09). All of them present extracellular regions (Fig 3A), functioning in growth factor binding (FLT1 and COL1A2), SMAD binding (COL5A2 and CO1A2) or extracellular matrix structural constituent (COL6A5, COL7A1 and COL1A2) (Fig 3B). The gene ontology (GO) biological process enrichment indicated that these proteins play a role in the vasculature (FLT1 and VEGFB), collagen fibril organization (COL1A2 and COL5A2), extracellular matrix organization (COL1A2, COL5A2 and COL7A1) and anatomical structure morphogenesis (FLT1, VEGFB, COL1A2, COL5A2 and COL7A1) (Fig 3C). Interestingly, 46.63% of CM-1 cases presented with rare variants in at least one of the four significant collagen genes and 9.55% harbor variants in multiple significant collagen genes. Moreover, 25.8% of CM-1 cases presented rare variants in *COL6A5* (S5 Table). These data suggest that the impact of these specific collagen genes on risk for CM-1 may be more universal compared to other genes.

**Table 2:** Candidate gene burden tests comparing the number of variants in CM-1 cases with public controls from gnomAD.

Candidate Gene	Number of SNPs passing filtering	Fisher exact 1-sided p-value	Odds Ratio (OR)	Lower Confidence Interval of OR	Upper Confidence Interval of OR
*PLOD1*	2	0.8149	0.794	0.265	1.785
*MSGN1*	1	+			
*COL3A1*	6	0.2935	1.362	0.633	2.519
*COL5A2*	12	**0.0001**	1.814	1.327	2.426
*COL7A1*	14	**0.0006**	2.424	1.501	3.69
*COL6A5*	23	**<0.0001**	1.99	1.463	2.648
*CDX1*	0	*			
*ADAMTS2*	5	0.2719	1.198	0.751	1.807
*TNXB*	48	0.9999	0.228	0.167	0.306
*VEGFA*	1	+			
*DSE*	4	0.8806	0.757	0.383	1.326
*COL1A2*	3	**0.0095**	8.273	2.303	20.678
*RDH10*	0	*			
*COL5A1*	7	0.0934	1.399	0.895	2.078
*NRP1*	1	+			
*VEGFB*	3	**0.0036**	7.436	2.467	16.884
*FLT1*	8	**0.001**	3.354	1.752	5.766
*HIF1A*	4	0.3037	1.509	0.568	3.178
*PGF*	1	+			
*ALDH1A2*	0	*			
*COL1A1*	4	0.0709	2.532	0.953	5.339

Significant p-values are indicated in bold.

+These genes only contained a single variant that surpassed our quality control filters and thus, were dropped from the burden analysis.

*These genes did not contain variants that surpassed our quality control filters and thus, were dropped from analysis.

When testing for an excess burden of functional variants between CTD+ compared to CTD- CM-1 cases, we identified two genes (*COL7A1*, *COL3A1*) where the burden of rare variants was significantly different between the CM-1 cases as a function of CTD status (*COL7A1* p = 0.0081 *COL3A1* p = 0.0193; Tables 3 and S3). CTD+ CM-1 cases had a significantly higher percentage of rare, functional variants in *COL7A1* and a significantly lower percentage of rare, functional variants in *COL3A1*. These associations remained significant when CM-1 patients who had a formal EDS diagnosis were excluded from the analysis (*COL7A1* p = 0.0025; *COL3A1* p = 0.0385; Table 3).

**Table 3:** Gene burden tests comparing CTD+ versus CTD- individuals.

Candidate Gene	N SNPs (N SNPs after removing EDS cases)	SKAT p-value	SKAT p-value excluding EDS cases
*PLOD1*	1 (1)	0.6325	0.5892
*MSGN1*	5 (5)	0.6314	0.6173
*COL3A1*	6 (6)	**0.0193**	**0.0385**
*COL5A2*	11 (10)	0.5262	0.5998
*COL7A1*	13 (13)	**0.0081**	**0.0010**
*COL6A5*	22 (21)	0.9536	0.9572
*CDX1*	0	*	*
*ADAMTS2*	5 (4)	0.6949	0.7651
*TNXB*	40 (38)	0.2784	0.2964
*VEGFA*	1 (1)	0.7303	0.7147
*DSE*	3 (3)	0.4226	0.4187
*COL1A2*	9 (9)	0.6671	0.7511
*RDH10*	0	*	*
*COL5A1*	11 (11)	0.4796	0.4058
*NRP1*	3 (3)	0.7976	0.7886
*VEGFB*	6 (4)	0.2104	0.2553
*FLT1*	9 (9)	0.1223	0.1337
*HIF1A*	4 (4)	0.5227	0.4971
*PGF*	1 (1)	0.2303	0.2147
*ALDH1A2*	0	*	*
*COL1A1*	13 (13)	0.4138	0.4937

Significant p-values are indicated in bold.

*These genes did not contain variants that surpassed our quality control filters and thus, were dropped from analysis.

## Discussion

In the present study, we provide evidence for an important role of collagens and other members of the extracellular matrix (ECM) in the pathogenesis of CM-1. We identified four variants in genes involved in the ECM (*COL6A5*, *COL7A1*), angiogenesis (*ADGRB3*) and adhesion junctions (*DST*) that cosegregated with CM-1 in a large Spanish family. Follow-up analysis of ECM genes in an independent cohort (186 CM-1 patients) identified an enrichment of rare functional variants in *COL7A1*, *COL5A2*, *COL6A5*, *COL1A2*, *VEGFB* and *FLT1* in CM-1 cases compared with controls. In our CM-1 cohorts, nearly half of unrelated CM-1 cases harbored rare variants in at least one of the significant collagen genes (*COL7A1*, *COL5A2*, *COL6A5*, *COL1A2*), suggesting that these genes may be particularly important for risk for CM-1. We also observed association between the presence of CTD symptoms among CM-1 patients with the occurrence of rare variants in *COL7A1* and *COL3A1*.

A role for genetic variation in collagens, and more broadly components of the ECM, in risk for CM-1 is perhaps not unexpected. Bone and ligaments are derived from connective tissue which is composed of the ECM (consisting of protein fibers such as collagens) and a variety of support cells. In particular, ligaments are composed of collagen (75% of the dry weight) and non-collagenous proteins (such as proteoglycans, glycoproteins and elastin) that are synthesized by fibroblasts. In bones, the connective tissue is highly vascularized and contains a specialized ECM mainly composed of collagen types I, III, and V that are synthesized by osteoblasts. The importance of collagens and other ECM genes in risk for EDS, a group of CTDs frequently comorbid with CM-1, is well known [6]. But there is also evidence supporting a role in CM-1 pathogenesis more directly. *COL2A1*, although not examined in this study, was found to be significantly upregulated in CM-1 patients with specific cranial morphometrics [16]. Moreover, the relationship between collagen and retinoic acid exposure has been observed in chondrocyte and osteoblast differentiation [20, 21] and over-expression of vitamin A can reduce bone mineral density [22] and even produce a Chiari-like phenotype [2]. Two SNPs (rs2899611 and rs6493979) of the *ALDH1A2* gene (an enzyme that catalyzes the synthesis of retinoic acid) have been previously associated with risk for CM-1 [15]. Thus, there is supportive evidence implicating collagens and associated pathways in CM-1. However, to our knowledge, this is the first report associating rare genetic variants in collagens and other ECM genes with the occurrence of CM-1.

Many of the individual genes associated with CM-1 in our study have known biologic roles that could impact CM-1 risk. Genetic variation in *COL6A5* influences bone mineral density in both mouse and human [23]. *COL7A1* is typically known for its association with epidermolysis bullosa, a blistering skin condition caused by abnormal collagen organization [24, 25]. However, COL7A1 is also downregulated in articular cartilage and subcondral bone in the setting of osteoarthritis [26]. COL5A2 and COL1A2 can participate in binding SMAD, a protein that is the main signal transducer for receptors of TGF-β. While SMAD has not been directly associated with CM-1, *TGFBR2* and *GDF6* are two other members of the TGF-β gene family that have been previously associated with CM-1 [12, 17]. Mutations in *COL3A1* have been associated with a vascular form of EDS [6], primarily characterized by risk for early death due to the rupture of major arteries [27]. *VEGFB* and *FLT1* are vascular genes, and it is known that vascularization and collagen is important for the bone mineralization process [28–30]. Vasculature is thought to serve as a guide for deposition of the mineralized bone matrix (osteoclast-secreted collagen type I), which helps define bone shape during embryonic development [29]. Alteration in the interactions between blood vessels and bone cells can lead to skeletal diseases such as craniofacial dysmorphology or idiopathic osteonecrosis [31].

### Collagens in co-occurring diseases

Classical CM-1 occurs because of a smaller and shallower posterior cranial fossa (PCF), as a consequence of a change in the length and slope of the basilar part of the occipital bone (basiocciput) [5, 32, 33]. However, other mechanisms can also lead to a small PCF, such as craniosynostosis (Crouzon’s syndrome or Apert syndrome). Craniosynostosis syndromes are frequently associated with mutations in fibroblast growth factor receptors (*FGFR2* or *FGFR3*) which play an important role in bone growth, particularly during embryonic development and are complexed with ECM components. Other conditions that can be comorbid with CM-1, such as osteogenesis imperfecta (OI) or scoliosis, are associated with alterations in the ECM [8]. OI is a genetic disorder predominantly affecting bone, and is characterized by bone fragility and abnormalities in skeletal, teeth, skin, and soft tissues, as well as joint hyperlaxity. OI is caused by alterations in collagen I, either as a consequence of haploinsufficiency of the genes (*COL1A1* or *COL1A2*) or due to genetic mutations in other genes related to collagen I biosynthesis and organization. Scoliosis, frequently associated with CM-1 [34, 35], is an abnormal curving of the spine due to connective tissue alterations and some forms of scoliosis are associated with abnormalities in collagens [36, 37].

Since each collagen is generally expressed in several different tissues, and is tightly associated with other ECM components, alterations in the collagen genes can result in widely overlapping clinical features [38]. For example, mutations in *COL1A2* can give rise to OI, EDS type VIIB, recessive EDS Classical type, idiopathic osteoporosis, and atypical Marfan syndrome. Mutations in *COL2A1* are associated with a wide range of clinical conditions including severe Ulrich congenital muscular dystrophy, hypochondrogenesis, and different forms of EDS to name a few [28]. Additional work will be needed to fully understand why mutations in the same gene result in different clinical presentations, but we hypothesize it is caused by allelic heterogeneity or by an incomplete penetrance resulting from compound inheritance of both, rare and common variants, at single or multiple loci as has been demonstrated for non-syndromic craniosynostosis and congenital scoliosis [39, 40].

### Study limitations and future work

CM-1 is highly clinically heterogeneous. In an attempt to reduce that heterogeneity, we took two different approaches. The first was to restrict to a more homogeneous demographic group of patients. The second was to subsequently stratify that group on the presence or absence of CTDs. However, there are many other ways to address the clinical heterogeneity in this patient population [8] and those may have yielded different findings than what we identified here.

With respect to the demographics of this population, we focused primarily on adult females of European descent. The Spanish family used in the exome sequencing study included pediatric and male subjects, but the unrelated CM-1 patients examined in the targeted sequencing analysis were exclusively Caucasian, adult females. CM-1 is more frequent in females, and thus, this restriction allowed us to achieve a reasonable sample size without concern for potential gender differences. The fact that we observed rare functional variants in *COL6A5* and *COL7A1* in both the Spanish family and the unrelated CM-1 cases suggests that these loci may not be unique to females with CM-1. It is possible that other mechanisms may be driving the gender differences. Recently Houston *et al*. observed a shorter PCF in males compared to females [41], underscoring the effect of gender on CM-1 traits. More work will be needed to disentangle the mechanisms leading to the gender differences. We also restricted the unrelated CM-1 cohort to adults, since it has been reported that the age of onset of CM-1 symptoms (pediatric versus adult) can produce different forms of the disease [42–44]. It is also important to note that the unrelated CM-1 cases utilized in the targeted sequencing were from the US, while the whole exome sequencing family was from Spain. However, both data sets have European origin, and variants in *COL6A5* and *COL7A1* were identified in both.

Although the evaluation of rare variants as a function of CTD status had considerably less statistical power than did the analysis of all the CM-1 cases versus public controls, we did observe evidence for some genes having a higher prevalence of rare variants as a function of CTD status. The association of *COL7A1* variants in the CTD+ subset is consistent with a role for collagens in connective tissue as described above. The association of *COL3A1* in the CTD- subset is more surprising. However, mutations in *COL3A1* have been typically associated with the vascular subtype of EDS [6]. Thus, it is possible that these individuals may not have received a diagnosis of EDS as a result of a more predominant vascular phenotype rather than joint involvement. More investigation will be needed to fully evaluate this association, including examination of a larger cohort of CM-1 patients. Nonetheless, the observation of different genetic etiologies as a result of CTD status among CM-1 patients is consistent with previous findings [12].

Our analytic approach for this study was to focus on rare variants in the coding regions of the genes, based on the assumption that rare, highly penetrant changes in these regions would produce an altered protein, resulting in CM-1. However, alterations in introns, regulatory regions, and long noncoding RNAs can also cause clinical presentations since these types of variants often impact gene expression. Future genetic studies in CM-1 should ideally focus on whole genome sequencing to ensure that these other types of mutations are fully evaluated in CM-1 etiology. We also focused the targeted sequencing analysis on genes belonging to a common biologic pathway. However, it is important to note that while *DST*, which was identified in the Spanish multiplex family, does not belong to the biologic pathway, there is recent work implicating this gene in a patient presenting with syringomyelia [45], a condition often comorbid with CM-1. Similarly, *ADGRB3*, also does not belong to the pathway that was sequenced in the US cohort, but it has been associated with intellectual disability and cerebellar atrophy [46]. Thus, we cannot exclude the possibility that the *DST* and *ADGRB3* variants contribute to CM-1 pathogenesis in the Spanish family. The finding of multiple variants in reasonable candidate genes in a single multiplex family is consistent with CM-1 being a complex genetic disorder, with possibly several variants contributing to the phenotype even within the same family. Taking a multi-omics approach to understanding CM-1 pathology may help disentangle the multiple candidate genes. Previous work has demonstrated evidence for genomic differences contributing to CM-1 subtypes [15, 16]. Thus, a more comprehensive analysis of genomic variation in CM-1 patients could shed light on relevant candidate genes and mechanistic processes.

The clinical data for the unrelated CM-1 patients in the targeted sequencing analysis was obtained from a variety of sources, including some self-report data. For the diagnosis of CM-1, medical record data were obtained, and MRIs, in the majority of cases, were available to confirm the diagnosis. Thus, misdiagnosis of CM-1 is not likely in this analysis. In contrast, the assignment of CTD status was rarely accompanied by a physician diagnosis and was primarily generated by self-report. As a result, the CTD classification used in this study has less precision. Nonetheless, that limitation would only affect the CTD status analysis, not the primary comparison of CM-1 cases to controls.

Future work should evaluate the effect of genetic variation on cranial morphometrics. Several studies have previously shown that CM-1 patients have unique cranial morphology [5], and also that genetic variation is associated with cranial morphology [15–17]. Cranial morphology is a quantitative phenotype which should yield more power to detect associations with genetic variants, and may also help distinguish different CM-1 subtypes. In addition, while this study has provided statistical evidence of association between rare functional variants and risk for CM-1, careful molecular functional analysis will be needed to fully define the manner in which the variants in these genes impact risk for CM-1.

## Conclusions

Rare functional variants within collagens and other related ECM genes are a risk factor for CM-1. CTD status may impact which collagen genes are involved, and thus may be a hallmark not only for clinical heterogeneity in CM-1 but also genetic heterogeneity. Follow-up studies in other CM-1 patients is warranted, but these findings may ultimately provide an opportunity for improved diagnosis and clinical management strategies in CM-1 patients.

## Methods

### Ethics statement

The present investigation was conducted according to the principles expressed in the Declaration of Helsinki and was approved by the Ethics Committee of the Vall d’Hebron University Hospital (Spain) and the institutional review boards of the University of Akron (OH, US) and Duke University School of Medicine (NC, US). All participants provided informed consent prior to their participation in the study.

### Subjects

#### Spanish multiplex family

A female, 30 years old Caucasian patient (index case, individual II.4) visited the Vall d’Hebron University Hospital (Spain). Due to the patient’s symptoms (cough headaches, neck pain, paresthesia in the upper and lower limbs, sensory loss, and impaired temperature sensation in the thorax, right arm and in both lower limbs), a cranial and complete spinal cord MRI was performed, and the neurosurgeon (MAP) diagnosed her with CM-1. The patient had 10mm of tonsillar herniation, retrocurved odontoid and cervical syringomyelia. Posterior fossa reconstruction was performed (extensive suboccipital craniectomy with a wide opening of the foramen magnum and C1 laminectomy; the dura mater was opened and closed watertight using a wide dural lyophilized allograft [Lyodura-S, Braun-Melsungen AG, Germany], wide enough to allow a spacious posterior fossa to be reconstructed with good results (new artificial cisterna magna formed with upward migration of the cerebellum and reduction of the syrinx [47]). Clinical interview and cranial MRI demonstrated CM-1 in four additional family members (Fig 1). The main clinical characteristics of the other affected members of the family included:

- **Individual II.1**: male, 38 years old. CM-1 (tonsillar herniation below the foramen magnum of 3 mm) without syringomyelia. Headaches.
- **Individual II.5**: male, 27 years old. Retrocerebellar arachnoid cyst, initially asymptomatic. Two years later, the patient reported symptoms that increased with stress: hemicranial headaches, visual alterations, and abnormal oral perceptions, which were attributed to migraine.
- **Individual III.1**: female, 9 years old, daughter of individual II.1. Frequent headaches. CM-1 with tonsillar herniation of 6 mm without syringomyelia.
- **Individual III.4**: male, 8 years old, son of individual II.4. CM-1 (tonsillar herniation below the foramen magnum of 4 mm) without syringomyelia.

All individuals studied had a normal ventricular size (Evans Index < 0.30) [48].

#### US caucasian cohort

Targeted candidate gene sequencing was performed in an independent cohort comprised of 186 unrelated adult American Caucasian females. Participants were recruited at two different sites: Ninety-four were recruited through the Chiari1000 project (https://chiari1000.uakron.edu) and ninety-two were selected from a larger family study of CM-1 ascertained at Duke University Medical Center [12]. Chiari1000 project participants shared pre-surgery MRI data, and those who had indicated interest in participating in future studies were contacted by email and invited to participate in the genetic study. Saliva samples were collected through the mail and extracted at Duke University Medical Center. Participants in Chiari1000 who ultimately provided a saliva sample were almost exclusively female and all Caucasian. As a result, only unrelated Caucasian females were selected from the Duke cohort in an effort to avoid potential confounding of racial, ethnic, and gender differences by site. Participants from the Duke family study were ascertained as part of a larger study to investigate the genetic basis of CM-1. Like the Chiari1000 subjects, Duke subjects were recruited primarily through self-referral across the US and provided medical records, including MRIs, which were used to confirm the diagnosis. Because Duke subjects were recruited primarily for the purpose of a genetic study, the majority had a positive family history of CM-1.

Clinical features of the Chiari1000 and Duke participants are described in Table 4. Among the 186 participants, 176 were diagnosed with CM-1 and ten were diagnosed with CM-1 and EDS. All 186 women presented with a minimum of 3mm cerebellar tonsillar herniation on MRI. Exclusion criteria included the following: 1) individuals thought to have a secondary form of CM-1 (e.g. due to a brain tumor, tethered cord syndrome, hydrocephalus, atlantoaxial assimilation, craniosynostosis or pseudotumor cerebri), 2) participants with another known genetic syndrome other than EDS (e.g. Marfan syndrome, Klippel-Feil syndrome, Neurofibromatosis, Growth hormone deficiency, abnormal pituitary, hyperostosis), and/or 3) participants that were not of non-Hispanic Caucasian ethnicity.

**Table 4:** Clinical characteristics of Chiari1000 and Duke subjects.

	Chiari 1000 (N = 94)		Duke (N = 92)	
	Number	%	Number	%
**CTD+**	28	29.79	53	57.61
**CTD-**	66	70.21	27	29.35
**Unknown CTD**			12	13.04
**EDS-**	89	94.68	87	94.57
**EDS+**	5	5.32	5	5.43
**Syringomyelia**	14	14.89	22	23.91
**No syringomyelia**	80	85.11	70	76.09

Previous work has demonstrated that, even in the absence of a formal CTD diagnosis, stratifying study participants on the presence (CTD+) or absence (CTD-) of CTD associated symptoms can improve the ability to detect a genetic signal [12]. Thus, we assigned CTD status based on a positive family history of CTD associated symptoms including self-reported hypermobility, a Beighton hypermobility score of 6 or greater [49], kyphosis, aneurysm, mitral valve prolapse, pectus excavatum, scoliosis, orthostatic hypotension, supraventricular tachycardia, heart valve disease, and/or heart murmur [12]. Study participants with a family history of at least one of these characteristics were classified as CTD+; all other participants were classified as CTD-.

### Brain Magnetic Resonance Imaging (MRI) protocol

MRI data for the Spanish family were acquired at Vall d’Hebron University Hosptial using a 1.5 T scanner (MAGNETOM Symphony or MAGNETOM Vision, Siemens, Erlangen, Germany) equipped with a circular polarized receiver head array coil. Sagittal conventional spin-echo T1-weighted sequences were obtained (TR 450–600 msec, TE 12–20 msec, acquisitions 2). All sequences were obtained with 4-5-mm section thickness and 0.1–0.3 interslice gap, with 144–256 × 256–384 matrix, and 196 × 230 mm field of view. MRI images were voluntarily provided by the participants recruited through the Chiari1000 project and the Duke family study, details about which have been described previously [12, 41]. In this analysis, the MRI’s were used to confirm the CM-1 diagnosis.

### DNA isolation and quantification

Genomic DNA from the Spanish family members was isolated from peripheral blood lymphocytes by the salting-out method [50]. Genomic DNA from the Chiari1000 participants was isolated from saliva using the Oragene DNA Self-Collection kit according to the manufacturer’s recommendations (DNA Genotek Inc. Ottawa, Ontario, Canada). For patients from the Duke cohort, genomic DNA was extracted from peripheral blood samples using Puregene reagents (Qiagen, Valencia, California). DNA quality was assessed by running a small amount of DNA on a 0.8% agarose gel. DNA concentrations were determined on a NanoDrop spectrophotometer (NanoDrop Technologies, LLC, Wilmington, DE) or using Quant-iT PicoGreen dsDNA Reagent (Thermofisher Scientific, Foster City, CA).

### Design, library preparation and next generation sequencing

#### Whole exome sequencing of Spanish multiplex family

A Covaris ultrasonicator (Covaris, Woburn, MA, USA) was used to shear 1μg of genomic DNA from individuals II.1, II.4, III.1 and III.4 resulting in fragments 300-400bp in size. Library generation was performed using the TruSeq DNA kit (Illumina, San Diego, CA, USA) following manufacturer’s instructions. Pools of six samples were subjected to whole exome capture applying Nimblegene SeqCap EZ Human Exome v3.0 (Roche NimbleGen, Madison, WI, USA) followed by sequencing on a HiSeq 2500 using the 100bp paired end protocol. The sequencing was performed at the Centre for Genomic Regulation in Barcelona (Spain). We obtained a coverage of at least 30X in > 80% of the enriched regions per sample.

#### Targeted sequencing of CM-1 cases

Amplicon targets were designed using DesignStudio, a web-based software tool (Illumina, San Diego, CA, USA). Initially, 23 genes were prioritized: those identified from whole exome sequencing analysis of the Spanish family, genes previously associated with CM-1 [15] or Elhers Danlos Syndrome (EDS) (https://ghr.nlm.nih.gov/condition/ehlers-danlos-syndrome#genes) and genes in pathways connected to those genes as identified using the STRING network database [51]. STRING was used to identify a potential protein-protein interaction (PPI) network among the candidate genes for the purpose of focusing our analysis on a defined molecular pathway. In total, 1144 amplicons were designed to capture the exonic regions of 21 genes. Libraries were prepared using the TruSeq Custom Amplicon Library Prep kit manufactured by Illumina, following the manufacturer’s recommendations. Sequencing of the libraries was performed on an Illumina MiSeq v2 using 150bp paired end reads by the Duke Center for Genomic and Computational Biology (GCB) at Duke University Medical Center.

### Whole exome sequencing

#### Alignment and variant detection

Sequencing data preparation and read alignment were performed according to Genome Analysis Toolkit (GATK) Best Practice recommendations provided by Broad Institute. In brief, sequencing reads were aligned to the hg19 reference genome using BWA-MEM [52]. Variants were called using GATK HaplotypeCaller (version 3.3) [53]. Initial quality control was performed using default thresholds in GATK and subsequently variants failing to meet those criteria were removed. Additionally, calls based on fewer than three reads (low coverage) and heterozygous calls with less than 30% of reads attributable to each allele (allele balance) were zeroed out. Only biallelic variants were considered for variant annotation.

#### Variant annotation and prioritization

Variants were annotated, filtered and prioritized using both functional annotations (population allele frequency, impact on protein function and conserved elements) and pedigree information. ANNOVAR [54] was used to annotate the variants, including: i) information on population allele frequencies based on: 1000 genomes 2015aug (Eur) [55], ExAC version 0.3 (NFE) [56] and esp6500siv2 (Exome Variant Server, NHLBI GO Exome Sequencing Project (ESP), Seattle, WA; URL: http://evs.gs.washington.edu/EVS/; date accessed: February, 2017)); ii) dbSNP version 147 with left normalization [Database of Single Nucleotide Polymorphisms (dbSNP). Bethesda (MD): National Center for Biotechnology Information, National Library of Medicine. (dbSNP Build ID: build 147)]; iii) dataset functional prediction dbnsfp30a which includes PolyPhen-2 [57], SIFT [58], MutationTaster [59], LTR [60], Mutation Assesor [59] and FATHMM [61]; and iv) information about evolutionarily conserved elements in mammalian species (phastCons20way mammalian) [62].

As the inheritance model in the pedigree appeared to be dominant, we performed variant prioritization based on this mode of inheritance and focused on exonic (non-synonymous and splicing) variants. In order to reduce the candidate variant list, we prioritized variants that had not been described previously (ie. were novel), presented with a minor allele frequency (MAF) <0.01, were predicted to be deleterious in at least two of the six functional prediction programs, and had a conservation site score over 0.9. Lastly, we checked the genotypes and the proportion of the reads for each individual using the Integrative Genomics Viewer (IGV) [63, 64] for the candidate variants in order to remove potential false positives, such as variants where the allele with less coverage had less than 30% of the reads at that locus. To ensure there were no sequencing errors, we confirmed the variants passing these filters using Sanger sequencing.

### Targeted sequencing

Raw sequencing reads from 21 targeted genes were processed identically to those from the Spanish multiplex family. We applied the same GATK quality filters, zeroed out low coverage calls and heterozygous calls with allele balance < 30%, however, we did not exclude variants based on MAF, predicted function, or conservation score. Although not used as a filter, variants were annotated using ANNOVAR to classify variants based on MAF thresholds and predicted function. Genotypes for rare (MAF<1%), functional exonic (nonsynonymous or splicing) variants were confirmed via Sanger sequencing.

### Sanger confirmation

Direct sequencing was used to confirm high priority candidate variants identified through WES and targeted sequencing. WES candidate variants were sequenced for all family members in order to evaluate co-segregation with disease. For the targeted sequencing, we confirmed variants in the genes that were significant in the burden tests, described below, in an effort to remove any false positives. Primers used were designed with the identified variant genomic target coordinates using PrimerZ [65] generating amplicons of 200-600bp in length. The sequences are detailed in S1 and S2 Tables. Amplicons were amplified by PCR (S3 and S4 Tables show the reaction conditions) and purified and sequenced either by Genewiz (Research Triangle Park, NC) for the targeted sequencing or the High Technology Unit (UAT) at Vall d’Hebron Institut de Recerca for the Spanish family. All sequences were analyzed using Sequencher v 5.4.5 (GeneTools, Ann Arbor, MI).

### Statistical analysis of targeted sequencing

Because we only sequenced CM-1 cases, we utilized controls from the Genome Aggregation Database (gnomAD; https://gnomad.broadinstitute.org/), restricting the gnomAD controls to those of non-Finnish European descent to more closely match the ethnic background of our CM-1 cases. Allele counts were compiled for CM-1 cases and non-Finnish European controls for each variant and were summed by gene to obtain a gene-based burden of risk alleles. Importantly, we required that the variant be present in the gnomAD database to be considered for further analysis. This was to avoid potential false positive associations that could be driven by variants detected only in the CM-1 cases. Chi-square tests were used to test for an increased burden of risk alleles (1-tailed test) in the CM-1 cases compared to controls. If cell counts were less than five, a Fisher’s exact test was employed. The gene-burden tests were performed with PROC FREQ in SAS version 9.4 (SAS Institute, Cary, NC) and were restricted to functional variants with MAF<5%. To test for gene-based differences in allele frequencies between CTD+ and CTD- CM-1 cases, we used SKAT-O [66], which combines a standard gene-burden test with the sequence kernel association test (SKAT) to maximize power. Comparisons by CTD status were based on variant functional annotation (only functional variants) and allele frequency threshold (MAF<5%) to be consistent with the case-control analysis.

## Supporting information

S1 Table Primers details used to confirm candidate variants identified in WES. (DOCX)

S2 Table Primers details used to confirm candidate variants identified in and targeted sequencing. (DOCX)

S3 Table PCR conditions used to validate variants identified in whole exome sequencing of Spanish family. (DOCX)

S4 Table PCR conditions used to validate variants identified in targeted sequencing of Duke and Chiari1000 cohorts. (DOCX)

S5 Table SNPs identified from targeted sequencing that contributed to gene burden tests. The listed variants were either a) significantly different between CM-1 cases and public controls or b) significantly different between CTD+ and CTD- CM-1 cases.
(DOCX)
