# Supplementary material for: Rare functional genetic variants in COL7A1, COL6A5, COL1A2 and COL5A2 frequently occur in Chiari Malformation Type 1
Source: PLoS One. 2021 May 11;16(5):e0251289. doi: 10.1371/journal.pone.0251289 (PMC8112708; doi:10.1371/journal.pone.0251289)
Supplement: S1 Table — (DOCX) [file pone.0251289.s001.docx]

| **Gene** | **Position (GRCh37/hg19)** | **Forward sequence (5’->3’)** | **Reverse sequence (5’->3’)** | **Amplicon size** |
| --- | --- | --- | --- | --- |
| *ADGRA2* | chr8:37693279 | AGACTGCACCCTGCAACTG | TGAGGCTTTTCTCCTCCAGA | 334 |
| *ADGRB3* | chr6:69348700 | GGCCAAATGACATAGGATGAA | TTGGAATTGCAGAGTTGCAT | 360 |
| *COL6A5* | chr3:130187662 | AGATTTGGAGGGTGCATCTG | AGGTGGAGGTCAGTGGAGTG | 371 |
| *COL7A1* | chr3:48623625 | TTCTACCAAGAACCCCCAGA | AAGGCTATCATGCAGCCACT | 272 |
| *COL15A1* | chr9:101816909 | CTCAGGAGGGCTTTGTTTGAGT | CTGTGGTCAGGGTGGAATGTC | 325 |
| *DST* | chr6:56507522 | ACACAATCCCACACCAATGA | TGCCACTTTTCACCGTTAGA | 293 |
| *ITIH5* | chr10:7628008 | TCTTTACCTCCAGTGGGTGACA | TCAGCCAAGTGCCCTTTACAT | 316 |
