# Supplementary material for: Rare functional genetic variants in COL7A1, COL6A5, COL1A2 and COL5A2 frequently occur in Chiari Malformation Type 1
Source: PLoS One. 2021 May 11;16(5):e0251289. doi: 10.1371/journal.pone.0251289 (PMC8112708; doi:10.1371/journal.pone.0251289)
Supplement: S2 Table — (DOCX) [file pone.0251289.s002.docx]

| **Gene** | **Position (GRCh37/hg19)** | **Forward sequence (5’->3’)** | **Reverse sequence (5’->3’)** | **Amplicon size** |
| --- | --- | --- | --- | --- |
| *COL3A1* | chr2:189851842 | ACCACATCTTTTGGCACACA | GCTACCATTTTCTTTTCCAACC | 358 |
| *COL3A1* | chr2:189855743 | GCACTTTCTAGTTTTCATGTTTTCC | TTCAAGTTGTCATTTTTCATGG | 440 |
| *COL3A1* | chr2:189858779 | TGATTCTTTTGACCACATTTCA | CAAAATGCAAAAGAAGCCAGT | 274 |
| *COL3A1* | chr2:189861933 | TGATTAGTTATTGCCCTTTGAGG | TGAGGTCCAGTTTCACCATTC | 259 |
| *COL3A1* | chr2:189863424 | TCTAGGGCTTGCCTGGTACA | TGCTCTATAACCTGCTGAAGAAGG | 513 |
| *COL3A1* | chr2:189864023 | TAGTTCCCACCCAGCTGTTC | CCTTGCAGACCAGGAGTACC | 400 |
| *COL5A2* | chr2:189899700 | AATTGCCCCCAGTTCTAGTG | CGCTTATGGAGACCACCAAT | 330 |
| *COL5A2* | chr2:189899755 | AATTGCCCCCAGTTCTAGTG | CGCTTATGGAGACCACCAAT | 330 |
| *COL5A2* | chr2:189901388 | AGCAAAACCTTGTTTATGCAAT | CCCTCTGTTAATTCTTTTGGAGA | 447 |
| *COL5A2* | chr2:189904234 | AGCCGCCTGATCTTCAGTAA | GACCACCCCCATCTAGTTACAA | 294 |
| *COL5A2* | chr2:189904265 | AGCCGCCTGATCTTCAGTAA | GACCACCCCCATCTAGTTACAA | 294 |
| *COL5A2* | chr2:189907975 | GCCAGGAAGACCCTGAAGAC | TTTTTCAGTGATCACCTACCATT | 312 |
| *COL5A2* | chr2:189909952 | CAAGCTTGAGGAATTTCATATTTTT | ACACATGGACATTTGCCTGA | 343 |
| *COL5A2* | chr2:189915371 | GAGACTCAAAAATTAATGTCCCTACA | ACGGATGAGCTGTTTTCACC | 445 |
| *COL5A2* | chr2:189918622 | GTCTCAAAGGAGCCTCCAAA | TCCTACACAGTGACCAATGTCC | 361 |
| *COL5A2* | chr2:189931144 | ACTGAACTGTGCAGGGAACC | GACTTCGTTAAATTAGGCTTCCAA | 311 |
| *COL5A2* | chr2:189940142 | GCATGTGGTGAGTGCTCTGT | AACTGAGTGAGGGTTATTGTCAGTT | 279 |
| *COL5A2* | chr2:190044330 | ATGGCAAACTCACCATCCTC | TAAAGCTGCATCGCTTGAAA | 296 |
| *COL7A1* | chr3:48607731 | CCCTGGATGGTGACATTAGG | GCCTCTGGACTCAAAGGAGA | 312 |
| *COL7A1* | chr3:48608574 | ACTCCCTCTTCCTCCTGTGG | AGCCCGTGTCTGAACTCTGT | 311 |
| *COL7A1* | chr3:48609817 | GACAGAGGTCAGGAGGCAAC | CCACAAGCCTGTTTCCAAAT | 446 |
| *COL7A1* | chr3:48612300 | GAGGGGGACTCTATGGAAGG | GTGTGCCATAACCCTGGAAT | 343 |
| *COL7A1* | chr3:48613087 | CCTGCAGGAGTGGAAGAGAG | TCAAGGTGGGTTGTTTAGGG | 333 |
| *COL7A1* | chr3:48614349 | GCACCCTGAGACCTCAGAAA | TGTTTGGGCTGATGTGAGTC | 264 |
| *COL7A1* | chr3:48619925 | TGTGGAGAGAGGATAGGAGCA | GGACCAGGAGTGGGACTTTT | 229 |
| *COL7A1* | chr3:48621017 | GATGGAGCAGTGGTTGATGA | CCCCTGGAAAGAAAGGAGAA | 264 |
| *COL7A1* | chr3:48622510 | AGGCCAGAGGCAGAACAGT | TGTTCCACCCCCAATAACAC | 292 |
| *COL7A1* | chr3:48623625 | TTCTACCAAGAACCCCCAGA | AAGGCTATCATGCAGCCACT | 272 |
| *COL7A1* | chr3:48624040 | ACCTCTCAAGGGTTCATCCA | CCTGCTCTGTCATCCTCCTC | 367 |
| *COL7A1* | chr3:48624478 | CCTATTGGGTGGTCAGGAGA | GAAGGGCAGAAAGGTGTGTC | 290 |
| *COL7A1* | chr3:48627789 | GAGAAGCAAGGGTCTGCAAG | GGTTGTGGTGTCAGATGCAA | 423 |
| *COL7A1* | chr3:48628920 | TGACAGTTCAGGGCTCAGTG | GCTCTCCAACAGGACCAGAG | 305 |
| COL6A5 | chr3:130098623 | TGGATGTGAAGGAAAATTGC | TTCAGCGTGGAAATTGTCTG | 397 |
| COL6A5 | chr3:130098639 | CTATCCAAGTTGGGAAATCCA | TTCAGCGTGGAAATTGTCTG | 274 |
| COL6A5 | chr3:130098653 | CTATCCAAGTTGGGAAATCCA | TTCAGCGTGGAAATTGTCTG | 274 |
| COL6A5 | chr3:130098666 | CATATGGCAGCAGAAGAGCA | TCATTCTGGAGCTTTTTCAGG | 262 |
| COL6A5 | chr3:130104078 | TTGACTGATGGGATGTCCAC | ACATCAGGCCTTGTCTCAGC | 301 |
| COL6A5 | chr3:130104153 | TGGGATGTCCACAGACAGAG | CCAGTCTTGGCATTTGGTTT | 382 |
| COL6A5 | chr3:130107482 | CAAGGTTCTAGTCAACCAGCTTC | CCAATCTGGGTTTTGTCTGC | 322 |
| COL6A5 | chr3:130107567 | TGCAGGATGTGAAGACATGA | CCCTTGGAGTGGGTGAAGTA | 312 |
| COL6A5 | chr3:130107599 | TGCAGGATGTGAAGACATGA | CCCTTGGAGTGGGTGAAGTA | 312 |
| COL6A5 | chr3:130107975 | GGAGTAGCGCAGGATGATGT | CTGGAGGTCACAGGCATTTT | 360 |
| COL6A5 | chr3:130114082 | ATGGCTCAATTTGGAAGCAA | TCTCCTATGCCCAAAACCAG | 296 |
| COL6A5 | chr3:130124469 | CATAGTGCCCATGTGCTTTG | GAAAATGAGGGAGGAAAGGAA | 257 |
| COL6A5 | chr3:130125119 | GGGATTGATGGACTTGATGG | TACCCCCTTGCTTCAATCAG | 344 |
| COL6A5 | chr3:130128898 | CACCTCTTATAGCTTGACCCAAA | TCCAAAATAATATCACTTGCCAGA | 381 |
| COL6A5 | chr3:130132401 | TAAGTGCCCAGTGTGAAAGC | TGATATGATTCCCAGCACCA | 285 |
| COL6A5 | chr3:130135644 | AGGCTGCATCAAGGAGTTGT | CCTAGACGATGGGTTGATGG | 389 |
| COL6A5 | chr3:130150310 | TTTGCAGGCCAGATTTCTTT | AAACCATGGCAACTCTTGCT | 335 |
| COL6A5 | chr3:130150464 | GAGCTTTAATAAAACACGGGACA | TGGCTGTTTGACCATTGCTA | 317 |
| COL6A5 | chr3:130150590 | AACTGTCCTGTGGGAGCAAG | TGGCTGTTTGACCATTGCTA | 253 |
| COL6A5 | chr3:130159330 | TGGGAAAGTTCCAGGAGAAA | CTTAGCCCTCAAAGCCATCA | 270 |
| COL6A5 | chr3:130159640 | TGGCTTTGAGGGCTAAGTGT | TCACCATTTTGTGACCAGGA | 374 |
| COL6A5 | chr3:130187662 | AGATTTGGAGGGTGCATCTG | AGGTGGAGGTCAGTGGAGTG | 371 |
| COL6A5 | chr3:130187718 | TGCCATGAGTATGGAAATGC | AGGTGGAGGTCAGTGGAGTG | 324 |
| *COL1A2* | chr7:94033892 | TTGGGAGAAAAGGAAAAGCA | CAGGTCCTTGGAAACCTTGA | 354 |
| *COL1A2* | chr7:94040384 | TTGCAGGGTAGTCCTGGTTC | CAAAAATGCAACTGTCAGCAA | 408 |
| *COL1A2* | chr7:94049737 | CAGATTCATCTTTGGTCCCATT | GGAAATAAACAGTTACAGCTCTGG | 329 |
| *VEGFB* | chr11:64003467 | GCAGGAAAACCAGTGAGGAC | CTCCTGTGCATCAGTCTCCA | 360 |
| *VEGFB* | chr11:64005068 | CCCCAGCTCTAGGGAAGACT | CCTTCCCAAACAAACACCAC | 331 |
| *VEGFB* | chr11:64005069 | CCCCAGCTCTAGGGAAGACT | CCTTCCCAAACAAACACCAC | 331 |
| *FLT1* | chr13:28885760 | AGACAAAATAGGGCAAAATGTTA | CCCAATCAATGCCATACTGA | 331 |
| *FLT2* | chr13:28897068 | AAAATTTCAGAGATGCATAGTATGTTG | CCCCTGTGCTAGTTCACGAT | 366 |
| *FLT3* | chr13:28964035 | AGAAAACAGCCTTTTTGTTGC | ATGTGCCAAATGGGTTTCAT | 339 |
| *FLT4* | chr13:28971113 | CCTGAGAGCAAGGATGAAGG | ACCTTGGTTGTGGCTGACTC | 250 |
| *FLT5* | chr13:28973215 | AAGCGCATATGAAGGCAAAT | AGCTGACACCCCAGCTTAAA | 369 |
| *FLT6* | chr13:29008200 | TTGGCTGCAAGCATAAGAGA | TTGATCCCTGATGGAAAACG | 254 |
| *FLT7* | chr13:29012432 | GCCCAGGTGTTTGTAAGGAA | GGGCCAAATTCAGAGACAAA | 389 |
| *FLT8* | chr13:29012444 | GCCCAGGTGTTTGTAAGGAA | GGGCCAAATTCAGAGACAAA | 389 |
