# Supplementary material for: Rare functional genetic variants in COL7A1, COL6A5, COL1A2 and COL5A2 frequently occur in Chiari Malformation Type 1
Source: PLoS One. 2021 May 11;16(5):e0251289. doi: 10.1371/journal.pone.0251289 (PMC8112708; doi:10.1371/journal.pone.0251289)
Supplement: S3 Table — (DOCX) [file pone.0251289.s003.docx]

| Temperature | Time | # cycles |
| --- | --- | --- |
| 95C | 5’ | 1 time |
| 95C | 30” | 9 times |
| 60C | 30” |  |
| 72C | 30” |  |
| 95C | 30” | 40 times |
| 56C | 30” |  |
| 72C | 30” |  |
| 72C | 10’ | 1 time |
| 4C | forever |  |
