# Supplementary material for: Rare functional genetic variants in COL7A1, COL6A5, COL1A2 and COL5A2 frequently occur in Chiari Malformation Type 1
Source: PLoS One. 2021 May 11;16(5):e0251289. doi: 10.1371/journal.pone.0251289 (PMC8112708; doi:10.1371/journal.pone.0251289)
Supplement: S5 Table — The listed variants were either a) significantly different between CM-1 cases and public controls or b) significantly different between CTD+ and CTD- CM-1 cases. (DOCX) [file pone.0251289.s005.docx]

| **Gene** | **% CM-1 cases present rare variants in this gene** | **Position (GRCh37/hg19)** | **SNP** | **Ref** | **Alt** | **CM-1 MAF** |
| --- | --- | --- | --- | --- | --- | --- |
| *COL3A1* | 3.9 | chr2:189851842 | rs111391222 | C | T | 0.002809 |
|  |  | chr2:189855743 | rs112185887 | G | A | 0.002841 |
|  |  | chr2:189858779 | rs200394946 | A | T | 0.002809 |
|  |  | chr2:189861933 | rs35795890 | C | A | 0.005682 |
|  |  | chr2:189863424 | rs1801183 | C | A | 0.002809 |
|  |  | chr2:189864023 | rs41263773 | G | A | 0.005618 |
| *COL5A2* | 19.7 | chr2:189899700 | rs141777954 | T | A | 0.002809 |
|  |  | chr2:189899755 | rs139229616 | C | T | 0.002809 |
|  |  | chr2:189901388 | rs140952583 | T | C | 0.002857 |
|  |  | chr2:189904234 | rs62184175 | G | C | 0.016854 |
|  |  | chr2:189904265 | rs770554435 | G | A | 0.002809 |
|  |  | chr2:189907975 | rs151187317 | C | T | 0.002825 |
|  |  | chr2:189909952 | rs146789395 | G | A | 0.002959 |
|  |  | chr2:189915371 | rs369072636 | G | A | 0.002809 |
|  |  | chr2:189918622 | rs116298748 | G | A | 0.027950 |
|  |  | chr2:189931144 | rs35852101 | A | G | 0.025281 |
|  |  | chr2:189940142 | rs76148000 | T | G | 0.048851 |
|  |  | chr2:190044330 | rs563606558 | T | C | 0.002809 |
| *COL7A1* | 10.1 | chr3:48607731 | rs199819125 | C | T | 0.002809 |
|  |  | chr3:48608574 | rs142059751 | A | T | 0.002809 |
|  |  | chr3:48609817 | rs759136317 | T | C | 0.002809 |
|  |  | chr3:48612300 | chr3:48612300 | T | C | 0.002809 |
|  |  | chr3:48613087 | rs200551525 | G | A | 0.002825 |
|  |  | chr3:48614349 | rs147633212 | C | T | 0.002825 |
|  |  | chr3:48619925 | rs144557024 | G | A | 0.002825 |
|  |  | chr3:48621017 | rs79378857 | G | A | 0.005618 |
|  |  | chr3:48622510 | rs375795047 | G | A | 0.002809 |
|  |  | chr3:48623625 | rs149011081 | C | T | 0.008427 |
|  |  | chr3:48624040 | rs2228563 | C | T | 0.002809 |
|  |  | chr3:48624478 | rs753761607 | C | T | 0.002809 |
|  |  | chr3:48627789 | rs116005007 | C | A | 0.011561 |
|  |  | chr3:48628920 | rs138791004 | C | T | 0.002809 |
| *COL6A5* | 25.8 | chr3:130098623 | rs140721388 | C | G | 0.005682 |
|  |  | chr3:130098639 | rs142949552 | C | T | 0.002841 |
|  |  | chr3:130098653 | rs151095015 | G | T | 0.005650 |
|  |  | chr3:130098666 | rs367852284 | C | T | 0.002825 |
|  |  | chr3:130104078 | rs771720914 | A | G | 0.002841 |
|  |  | chr3:130104153 | rs149308947 | G | A | 0.005618 |
|  |  | chr3:130107482 | rs9882852 | A | C | 0.005848 |
|  |  | chr3:130107567 | rs144238271 | T | G | 0.002924 |
|  |  | chr3:130107599 | rs558128400 | T | G | 0.002825 |
|  |  | chr3:130107975 | rs16827168 | A | G | 0.016854 |
|  |  | chr3:130114082 | rs1353613 | C | G | 0.005618 |
|  |  | chr3:130124469 | rs77730506 | G | A | 0.002809 |
|  |  | chr3:130125119 | rs756847020 | A | G | 0.003049 |
|  |  | chr3:130128898 | rs190625267 | A | G | 0.002809 |
|  |  | chr3:130132401 | rs190283135 | C | T | 0.002809 |
|  |  | chr3:130135644 | rs76864445 | C | A | 0.008671 |
|  |  | chr3:130150310 | rs61744488 | A | C | 0.047753 |
|  |  | chr3:130150464 | rs754763520 | G | A | 0.002809 |
|  |  | chr3:130150590 | rs1461357665 | G | T | 0.002809 |
|  |  | chr3:130159330 | rs2201717 | C | T | 0.002809 |
|  |  | chr3:130159640 | rs572630219 | T | G | 0.002924 |
|  |  | chr3:130187662 | rs115375867 | G | T | 0.002809 |
|  |  | chr3:130187718 | rs930348677 | G | A | 0.002809 |
| *COL1A2* | 1.7 | chr7:94033892 | rs189557655 | C | T | 0.002809 |
|  |  | chr7:94040384 | rs764780528 | G | A | 0.002809 |
|  |  | chr7:94049737 | rs189374343 | A | T | 0.002809 |
| *VEGFB* | 1.7 | chr11:64003467 | rs111555072 | C | G | 0.005618 |
|  |  | chr11:64005068 | rs748964141 | A | C | 0.002809 |
|  |  | chr11:64005069 | rs768144934 | C | T | 0.002809 |
| *FLT1* | 6.2 | chr13:28885760 | rs140861115 | G | A | 0.008621 |
|  |  | chr13:28897068 | rs35549791 | T | C | 0.002809 |
|  |  | chr13:28964035 | rs142425372 | T | C | 0.002809 |
|  |  | chr13:28971113 | rs148479824 | T | C | 0.005618 |
|  |  | chr13:28973215 | rs770902566 | C | T | 0.002809 |
|  |  | chr13:29008200 | rs1014160326 | C | T | 0.002809 |
|  |  | chr13:29012432 | rs558999317 | G | T | 0.002809 |
|  |  | chr13:29012444 | rs755750116 | G | A | 0.002809 |

MAF=minor allele frequency.
